# Current Immunotherapy Treatments of Primary Breast Cancer Subtypes

**DOI:** 10.3390/biomedicines12040895

**Published:** 2024-04-18

**Authors:** Savannah R. Brown, Emilie E. Vomhof-DeKrey

**Affiliations:** 1 Department of Pathology, School of Medicine and the Health Sciences, University of North Dakota, Grand Forks, ND 58202, USA; emilie.dekrey@und.edu; 2 Department of Surgery, School of Medicine and the Health Sciences, University of North Dakota, Grand Forks, ND 58202, USA; 3 Department of Biomedical Sciences, School of Medicine and the Health Sciences, University of North Dakota, Grand Forks, ND 58202, USA

**Keywords:** breast cancer, hormone receptor, HER2, TNBC, immunotherapy, checkpoint inhibitors, clinical trials

## Abstract

Breast cancer receives the most funding when compared to any other cancer type, according to a global study conducted by *The Lancet*. Nevertheless, this malignancy remains the most diagnosed cancer among women and relies heavily on a neoadjuvant treatment regimen of chemotherapy and targeted therapy. After standard treatment, 25–30% of breast cancer patients still develop disease recurrence and must undergo cytoreductive debulking surgery followed by intensive chemotherapy. An array of targeted therapies are currently being utilized and developed to alleviate negative side effects, eradicate cancer growth, and diminish disease recurrence. Immunotherapy is a promising cancer therapy that upregulates one’s immune system to stimulate a therapeutic effect and is utilized for cancer management among other ailments such as immunodeficiencies, hypersensitivity reactions, autoimmune diseases, inflammatory disorders, tissue and organ transplantation, and infectious diseases. This review highlights the five primary subtypes of breast cancer, provides a brief history of immunotherapy, evaluates the current landscape of treating breast cancer with immunotherapy, analyzes selected ongoing or recently completed immunotherapy clinical trials for hormone receptor-positive, HER2-enriched, and triple-negative breast cancer, and examines future trends for the treatment of breast cancer with immunotherapeutic techniques. This review provides a formal summary categorized by breast cancer subtype rather than types of immunotherapeutic treatment.

## 1. Breast Cancer Classification

Breast cancer is the second leading cause of death for women and the most diagnosed malignancy worldwide despite being the most highly funded cancer type [1,2,3]. Although breast cancer is technically classified as one disease, it is difficult to treat due to the numerous subtypes and varying treatment regimens. The main subtypes of breast cancer are (1) luminal A, (2) normal-like, (3) luminal B, (4) HER2-enriched, and (5) triple negative (TNBC) [1,4,5]. Luminal A and normal-like breast cancer share similar characteristics, although they differ in overall genetic composition, and normal-like resembles a healthy breast profile [1,5]. Differentiating between luminal A and normal-like breast cancer poses a challenge due to the positive expression of estrogen receptor (ER) and progesterone receptor (PR), negative expression of human epidermal growth factor 2 (HER2), and low levels of Ki67, a common proliferation marker for human tumor cells, in both subtypes [6]. Luminal B breast cancer also has high levels of ER and PR but is typically HER2-positive and exhibits high Ki67 expression, resulting in faster cell growth [1,2,5]. It is important to note that over 70% of breast cancer patients have elevated ER levels [1,4]. Furthermore, there is a population of breast cancer patients that have amplification or overexpression of the human epidermal growth factor receptor 2 (HER2) and are classified as HER2-enriched [1,4,5,6]. These individuals are negative for both ER and PR, have faster cell growth than both the luminal and normal-like subtypes, and correlate with approximately 20% of breast cancer diagnoses [1,2,6]. TNBC is uniquely aggressive and has a poor prognosis due to the lack of ER, PR, and the HER2 oncogene [1,2,6,7]. Additionally, TNBC tumors originate from basal myoepithelial cells, which are absent in luminal tumors and exhibit stronger survival with increasing migration, invasion, and tumorigenicity [5,8]. This subtype is highly associated with BRCA1 mutations and occurs more often in women under the age of 40 [1,6,7]. A summary of the five common breast cancer subtypes can be found in Table 1.

**Figure 1:**
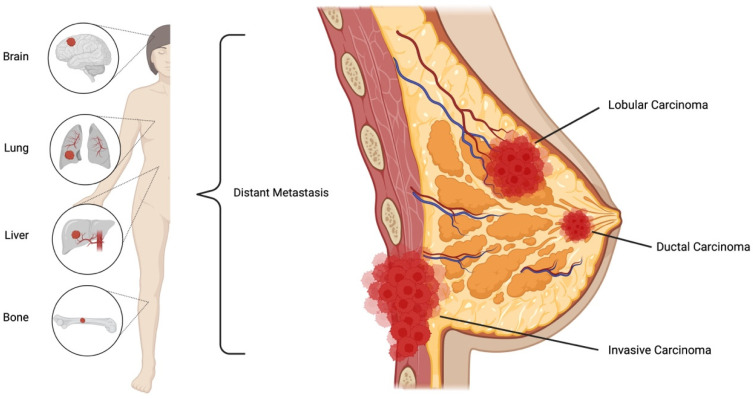
Common breast cancer carcinoma and distant metastasis. The majority of breast cancers develop into lobular or ductal carcinomas that can spread through lymph nodes to distant sites. Created using BioRender [13].

Breast cancers largely arise from the ductal or glandular tissue; therefore, the two most prevalent types of breast cancer are invasive ductal carcinoma and invasive lobular carcinoma [9,10,11]. Although breast cancer often develops from the lobules or ducts, the disease often spreads through lymph nodes or blood vessels, resulting in a metastasized—and often fatal—form of cancer [9,10,12]. Breast cancer most commonly spreads to distant organs, such as the brain, lungs, liver, and bones, as depicted in Figure 1 [2,5,9]. Along with lobular and ductal carcinomas, there are numerous other forms of breast cancer, such as inflammatory breast cancer, medullary, and Paget’s disease, but they are less common [5,9,10]. Additionally, ductal carcinoma in situ (DCIS) is a non-invasive form of cancer where the cancerous cells have not spread to breast tissue but rather reside within the ducts [5,9,10]. DCIS is often discovered during routine screening and accounts for 20–25% of all breast cancer diagnoses in the United States [9,12].

## 2. Introduction to Common Breast Cancer Therapies

Due to surgical and systematic advancements in cancer therapy, the standard care of treatment for breast cancer has shifted to a neoadjuvant therapy regimen comprising chemotherapy and targeted therapy, followed by surgery and radiation if the malignancy returns [1,2,4,6]. Approximately 25–30% of breast cancer patients experience cancer recurrence, often accompanied by metastases to the bone, liver, lungs, brain, and other sites [2,14]. Determining the best course of action for the management of breast cancer is a multifactor process that assesses the stage, hormonal responses, gene mutations, growth rate, and age of the individual [1,2,6,7]. Monotherapy, or treatment with a single agent, was the standard treatment of care for many years and included the following therapies: radiotherapy, cytotoxic chemotherapy, endocrine therapy, immunotherapy, and target therapy [1,2,15,16]. While this therapy worked for some individuals, many patients developed drug resistance, weakness, and relapse following preliminary treatment, along with negative side effects of these remedies [1,2,4,16]. Metastatic and TNBC patients had a particularly difficult time with monotherapy due to the aggressiveness and metastatic nature of these disease subtypes [1,2,4,7,16]. There is a critical need for the development of precise personalized treatment for the management of breast cancer. To combat these issues, combination therapy has been utilized since 1965 [1,16,17]. Two or more treatments are used simultaneously, leading to more effective results and lower rates of toxicity compared to a single agent alone [1,16]. Furthermore, using data analysis, such as nanotechnology, DNA sequencing, and computational analysis, we can optimize a personalized medicinal approach to treat breast cancer [1]. While strides have been made in the management of breast cancer, there is still a critical need for developing precise personalized treatment plans.

## 3. The History of Immunotherapy

To understand and broaden the landscape of immune-based therapy, we will briefly discuss the work that has previously been completed. We are inclined to believe that immunotherapy is a modern triumph for cancer treatment; however, engaging the immune system for the use of treatment dates back to the 1700s, with three documented cases of individuals utilizing inoculation to prevent smallpox in 1718, 1765, and 1796 [17,18,19]. These individual cases founded the field of vaccinations but were each severely overlooked due to resistance and skepticism from the scientific community [17,18,19]. Although inoculations were not directly used for the treatment of cancer at this time, they provided a foundation for treating disease through the stimulation of an individual’s immune system. The theory of an immune-based approach to treating disease was overlooked until two German scientists, Fehiesien and Busch, individually observed tumor regression following bacterial infection in 1866, and this was the first time in history that the immune system was shown to treat cancer [17,18,20]. It was not until 1883 that Fehiesien identified these bacteria as *Streptococcus pyogenes* [17,18,20]. Further, in 1891, William Coley attempted to cure bone cancer by injecting individuals with combinations of inactivated and live strands of *Streptococcus pyogenes* and *Serratia marcescens*, creating the first immune-based therapy for cancer treatment and coining the term “Father of Immunotherapy” [17,18,19,21,22]. Even though Coley had tremendous success with over 1000 documented cases of complete remission or regression in cancer patients, his work was criticized due to harsh side effects, causing his work to be ignored for years [17,18,19,21,22].

**Figure 2:**
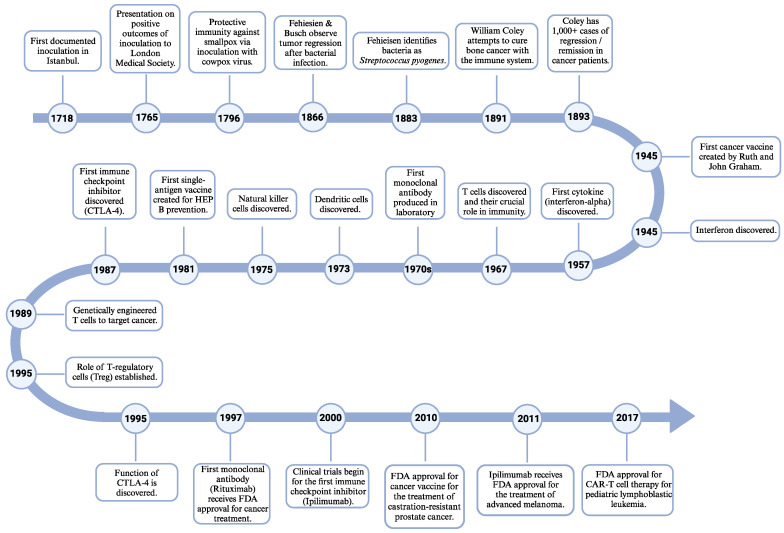
Major developments in immunotherapy. Depicted timeline highlighting significant milestones in immunology and immunotherapy treatment between the years 1718 and 2017. Created using BioRender [13].

The 1900s were an explosive time for immune-oncolytic therapeutic development. In 1945, interferon was discovered [17,23], and the first cancer vaccine was created by Ruth and John Graham [17,19]. In 1957, the first cytokine, interferon-alpha, was discovered [17,23,24]. Further, in 1967, the role of T cells in immunity was confirmed [17,25], followed by the discovery of dendritic cells in 1973 and natural killer cells in 1975 [17,19,23,26]. In 1981, the first vaccine with a single cell surface antigen was introduced for the prevention of hepatitis B [17,19]. Although this discovery was not specifically for the treatment or prevention of cancer, it was monumental because it established the groundwork for the development of cancer vaccines throughout the 2000s [17,21,27,28,29]. One of the most notable cancer vaccines received FDA approval in 2010 for the treatment of castration-resistant prostate cancer [17,21,27,30]. These studies acted as a catalyst for the development of an autologous HER2/neu (ErbB) experimental vaccine for the treatment of HER2-enriched breast cancer [17,27,31,32]. The first documentation of genetically engineered T cells to target cancer was in the year 1989 [17,33,34]. It was not until 1995 that the role of T regulatory cells (Treg) was established. This discovery was the springboard for chimeric antigen T-cell (CAR-T) therapy [17,19,20,35]. The first CAR-T therapy received FDA approval in 2017 for the treatment of pediatric lymphoblastic leukemia [17,20,36,37,38]. Antibody-based treatment is the most established immunotherapy to date [1,16,24,39]. Throughout the 1970s, the first monoclonal antibody was produced in the laboratory by Milstein and Köhler [1,17,21,40]. This was achieved by fusing lymphocyte and myeloma antibody-secreting cell lines together [17,21,40]. It was not until 1997 that the first monoclonal antibody, Rituximab, received the stamp of FDA approval for cancer treatment [17,21,41]. Additionally, in the realm of antibody-based therapies, cytotoxic T lymphocyte antigen (CTLA-4) was the first immune checkpoint inhibitor to be discovered in 1987, but its function was not established until 1995 [17,24,42,43,44]. Clinical trials for the first checkpoint inhibitor, ipilimumab, began in the year 2000 and received FDA approval for the treatment of advanced melanoma in 2011 [17,19]. For reference, a timeline of the major developments in immunotherapy is depicted in Figure 2.

The field of immunology is continuously developing and moving at a rapid pace. Targeted immune-based therapy provides additional treatment plans due to its ability to stimulate the patient’s immune system while achieving a therapeutic effect [16,17,45]. Published studies have shown that immunosuppressed individuals have an increased risk of developing cancer [17,46,47,48]. Additionally, while administered, standard chemotherapeutics act as immunosuppressants [1,17,47]. There are numerous documented cases that show the formation of new tumors in patients being treated with chemotherapy, highlighting the vast importance of immune stimulation during cancer treatment [17,46]. Therefore, the continued development of immune-oncolytic therapeutics is essential.

## 4. Immunotherapy for the Treatment of Breast Cancer

For the treatment of breast cancer, immunotherapy is a therapeutic technique that is commonly employed [39,49,50]. Immunotherapy is not a single treatment but rather a group of treatments used to upregulate or downregulate the immune system to achieve a therapeutic effect in immunologically mediated disorders, including cancer [51]. Tumors exhibit immunosuppressive characteristics, contributing to the microenvironment surrounding the tumor and advancement of the malignancy [17,18,45]. Re-engaging the immune system using immunotherapy can counter these effects and work to diminish tumor growth [17,52,53]. In breast cancer, the primary immunotherapy types are (1) antibody-based treatment, (2) cytokine treatment, (3) immune checkpoint inhibitors, such as PD-1, PD-L1, and CTLA-4, (4) adoptive T-cell therapy, and (5) anti-cancer vaccines, which are described in more detail below [16,39,49,50].

**Figure 3:**
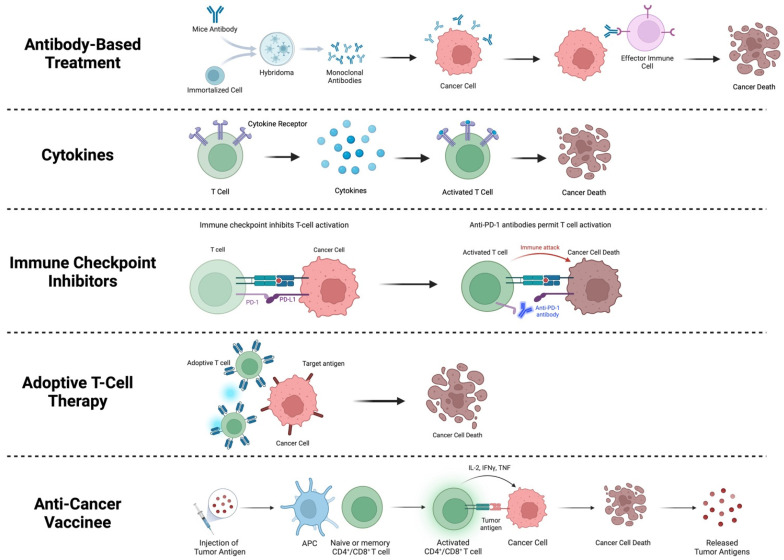
Immunotherapies used in breast cancer treatment. Brief description of antibody-based treatment, use of cytokines, immune checkpoint inhibitors, adoptive T-cell therapy, and anti-cancer vaccinees is depicted. Created using BioRender [13].

(1) Antibody-based treatment is conducted by the use of monoclonal antibodies, also known as mAbs [16,54]. mAbs recognize specific proteins in cancer cells to facilitate an immune response. Once the cancer cell is targeted, it promotes the activation of T cells, natural killer cells, and macrophages, which, in turn, produce a cytotoxic antitumor effect in patients [16,54]. mAb treatment has been prominent for the treatment of HER2-positive breast cancer, with two first-line FDA-approved mAbs trastuzumab and petuzumab [16,54]. (2) Utilizing cytokines for the treatment of cancer has been employed since the late 1950s [17,23,24,51]. Cytokines are regulators of both innate and adaptative immunity, which allow the immune system to correspond over short distances and can promote or inhibit cancer growth [51,55,56]. (3) Immune checkpoint inhibitors are designed to block checkpoint proteins, such as programmed cell death-1/programmed cell death ligand-1 (PD-1/PD-L1) and cytotoxic T-lymphocyte-associated protein 4 (CTLA-4), from binding with their partner proteins [16,39]. This blockage results in the activation of the T-cell response, subsequently killing the cancer cells [16,39]. (4) The goal of adoptive T-cell therapy is to endorse a cell-based anti-tumor immunity in cancer patients through the transfer of lymphocytes or other immune cells [16,37]. There are a variety of adoptive T-cell therapies, which include TIL-based therapy, chimeric antigen receptor (CAR) therapy, engineered T-cell receptor (TCR) therapy, dendritic cell-based therapy, and natural killer cell-based therapy [16,37]. (5) Anti-cancer vaccines intend to encourage an antigen-specific T-cell-based activation of the immune system with the goal of eliminating cancer cells [16,57]. Anti-cancer vaccines for breast cancer are separated into two categories: HER2-associated antigen vaccines and non-HER2 targeting vaccines [16,57]. These treatments, however, do require further development before becoming a standardized therapy [16]. A summary of these immunotherapies is summarized in Figure 3.

There are currently 13,733 clinical trials ongoing for breast cancer treatment [58]. Of these trials, only a mere 3%, or 434 trials, are utilizing immunotherapy [56]. The use of immune checkpoint inhibitors has been a successful strategy to modulate T-cell activation and T-cell tolerance to encourage immune homeostasis [16,17,39]. Clinical trials can be broken down into three phases, which are designated as (I) to test if a new treatment is safe, (II) to evaluate if the ailment responds better to a new treatment, and (III) to assess if a new treatment is better than the standard treatment [59]. Below are selected immunotherapy clinical trials for the treatment of hormone receptor-positive, HER2-enriched, and triple-negative breast cancer. Additionally, the I-SPY2 clinical trial will be highlighted.

While we recognize that these trials have not been completed, they emphasize the future direction that immunotherapy-based treatments are moving. Each trial exhibits novel combinations of immunotherapeutics and standardized chemotherapy in an effort to increase efficiency. Understanding these trials will provide insight into which immunotherapy trials are beneficial, detrimental, or need adjusting. Interestingly, the I-SPY2 clinical trial is not only testing novel therapeutics but is also evaluating a novel platform for clinical trials.

### 4.1. Hormone Receptor-Positive Breast Cancer

Breast cancer that is positive for estrogen receptor (ER) and progesterone receptor (PR) can be classified as hormone receptor (HR)-positive breast cancer [2,4,6,60]. Due to the presence of ER and PR, additional treatment options, such as endocrine therapy, are available [1,2,6,15,60]. Aromatase inhibitors (letrozole, anastrozole, and exemestane) and anti-estrogens (tamoxifen and fulvestrant) are the primary forms of endocrine therapy and are readily available for treatment [1,2]. Although treatment for HR-positive breast cancer may appear as the best course of action, this treatment causes infiltration of immune cells into the tumor microenvironment, resulting in immunomodulatory effects [1,61]. Numerous clinical trials for HR-positive breast cancer utilize neoadjuvant chemotherapy or hormone therapy in combination with immunotherapeutic agents.

The recently completed randomized phase I trial (NCT04148937) for advanced cancer, including HR-positive breast cancer, evaluated LY2475070 (CD73 inhibitor) administered alone or in combination with pembrolizumab [56]. Although the study has been completed, the final results have not been posted yet [58]. An ongoing phase II trial (NCT02971748) is being conducted for patients receiving hormone therapy who did not initially achieve a pathological complete response to chemotherapy [58]. The safety and toxicity profile will be evaluated for patients who receive a combination of pembrolizumab and hormone blockers before or during radiation treatment [58]. A phase II trial (NCT04243616) for the treatment of invasive HR-positive HER2-negative breast cancer with cemiplimab added to neoadjuvant chemotherapy with paclitaxel, carboplatin, doxorubicin, or cyclophosphamide is currently ongoing [58]. The pathological complete response will be assessed in all 36 patients treated with neoadjuvant chemotherapy and cemiplimab [58].

Currently, 400 patients are participating in a randomized phase II trial (NCT02491697) for the treatment of advanced breast cancer undergoing treatment of capecitabine monotherapy with or without DC-CIK immunotherapy [58]. Overall survival (OS) of 1 year and disease-free survival (DFS) of 6 months will be evaluated along with the side effects and clinical benefit response when comparing monotherapy to the combination of capecitabine and DC-CIK [58]. These findings and ongoing studies highlight the importance of expanding the field of immunotherapy for HR-positive breast cancer patients [58]. Table 2 highlights additional selected immunotherapy clinical trials for the treatment of HR-positive breast cancer.

### 4.2. HER2-Enriched Breast Cancer

Breast cancer patients within the HER2-enriched population lack hormone receptors, excluding these individuals from the option of endocrine therapy. Although they do not qualify for hormone therapy, monoclonal antibody immunotherapies that target HER2, such as trastuzumab, trastuzumab-DM1, and petuzumab, are used to treat HER2-enriched patients [1,21,62,63,64]. Trastuzumab is an FDA-approved first-line treatment for HER2-enriched breast cancer that elicits an immune response by activating macrophages, T cells, and natural killer cells to produce a cytotoxic anti-tumor cellular effect [1,21,63,64]. Numerous clinical trials are ongoing to evaluate additional innovative immunotherapies, such as CAR-T therapy, dendritic cell therapy, natural killer cell therapy, and others.

Phase I trial (NCT03740256) is evaluating the effectiveness and safety of HER2-specific CAR-Ts in combination with CAdVEC, an oncolytic adenovirus [58]. While it is believed that the combination of the HER2 CAR-Ts and CAdVEC oncolytic virus will have a synergistic relationship, the level of dosage and safety remains obscure [58]. The overall response rate (ORR), disease control rate (DCR), progression-free survival (PFS), OS, and number of treatment-related adverse events will be assessed [58]. Furthermore, in the four-part phase I/II clinical trial (NCT04278144), the novel immune-stimulating antibody conjugate (ISAC), BDC-1001, is being evaluated alone and in combination with nivolumab for the treatment of HER2-positive cancers, including breast cancer [58]. Dose escalation of BDC-1001 as a single agent and in combination with nivolumab will be examined along with the incidence of adverse and serious adverse events, dose-limiting toxicities, potential immune-related toxicities, the maximum tolerable dose (MTD), ORR, DCR, and PFS [58]. Although this is an ongoing trial, preliminary results have been reported, which indicate that evidence of clinical activity has been observed, including patients previously treated with anti-HER2 therapies [31]. Additionally, BCD-1001, administered at the dose of 5 mg/kg, has been well tolerated by patients, and dose escalation is currently ongoing [31]. These results permit advancement to the second phase of this study, which will evaluate the combination of BCD-1001 and nivolumab [31]. Additional selected immunotherapy clinical trials for the treatment of HER2-positive breast cancer can be found in Table 3.

### 4.3. Triple-Negative Breast Cancer

Due to the lack of hormone receptors and the absence of HER2, targeted treatment options remain extremely limited for TNBC patients, resulting in an aggressive phenotype with a poor prognosis [2,7]. Immunotherapy has initiated a transformation in treatment opportunities for TNBC patients, providing these individuals with the opportunity to seek additional treatment outside standard chemotherapy. Within the field of immunotherapy, TNBC is one of the most studied malignancies to date [1,49,50]. Treatment with immune checkpoint inhibitors, specifically anti-PD-1/PD-L1, is the primary focus for TNBC therapy. Below are selected ongoing TNBC immunotherapy clinical trials.

The recently completed KEYNOTE-355 double-blind phase III clinical trial (NCT02819518) evaluated 847 locally recurrent inoperable or metastatic TNBC patients who were not previously treated with chemotherapy. The study aimed to evaluate the safety of the PD-L1 inhibitor pembrolizumab in combination with paclitaxel, gemcitabine, carboplatin (566 patients), or a placebo (281 patients) [2,65]. Preliminary results indicate that the combination of pembrolizumab and chemotherapy resulted in significantly longer progression-free survival and a larger reduction in risk of disease progression or death when compared to the treatment of chemotherapy alone [2,65]. It is important to note that adverse side effects, such as anemia, neutropenia, and nausea, were not increased in the presence of pembrolizumab [2,65]. Additionally, the phase II KEYLYNK-009 trial (NCT04191135) is a randomized, open-label study for the treatment of TNBC. This study is evaluating the efficiency of olaparib, the poly ADP ribose polymerase inhibitor that blocks single-stranded DNA repair in combination with pembrolizumab, when compared to olaparib plus pembrolizumab following first-line chemotherapy, consisting of carboplatin, gemcitabine, and pembrolizumab [1,56]. It is hypothesized that the combination of olaparib and pembrolizumab will have a better outcome when compared to the study counterpart for both progression-free survival and overall survival [58]. The study is expected to be completed by September 2024. Furthermore, the P-RAD phase II randomized clinical trial (NCT04443348) aims to determine the effectiveness of pembrolizumab with or without radiotherapy, followed by adjuvant chemotherapy, radial therapy, or breast and axillary surgery [58]. In total, 120 patients are currently participating in the trial, which is expected to conclude in December 2024 [56].

The randomized phase II TONIC trial (NCT02499367) will evaluate TNBC patients treated with neoadjuvant therapy consisting of radiation therapy, low-dose doxorubicin, cyclophosphamide, and cisplatin, followed by the PD-1 inhibitor nivolumab [58]. Progression-free survival, overall response rate, clinical benefit rate, and toxicity profiles will be assessed [58]. The recently completed phase III IMpassion030 trial (NCT03498716) assessed the safety, efficiency, and pharmacokinetics of the anti-PD-L1 antibody atezolizumab in combination with paclitaxel, cyclophosphamide, and dose-dense doxorubicin or dose-dense epirubicin versus chemotherapy treatment alone in 2203 stage II and III TNBC patients [66]. Completion of this trial has received novel approval for treatment with atezolizumab in combination with nab-paclitaxel, becoming the first immunotherapy used as a first-line treatment for breast cancer [66]. Patients must be PD-L1-positive at the primary tumor or metastatic site to qualify for this therapy. The IMpassion130 trial has propelled new research to be conducted for novel predictive biomarkers and combinations with atezolizumab for the treatment of TNBC. Table 4 depicts additional selected ongoing clinical trials for the treatment of TNBC with immunotherapeutic agents.

### 4.4. I-SPY2 Trial: Neoadjuvant and Personalized Adaptive Novel Agents to Treat Breast Cancer

In the field of breast cancer, the I-SPY2 (investigation of serial studies to predict your therapeutic response with imaging and molecular analysis 2, NCT01042379) clinical trial must be highlighted. The purpose of the study is to assess the efficiency of novel drugs in combination with standard chemotherapy to advance the field of personalized medicine [67,68]. There are currently 5000 breast cancer patients enrolled in the I-SPY2 trial with 40 different treatment combinations [16,67,68]. The study has been ongoing since January 2010 and is expected to be completed in December 2030. Both the treatments and the clinical trial structure itself are novel. Rather than following the standard clinical trial model, the I-SPY2 trial follows a novel multi-agent adaptive model [69]. This “platform trial” allows for the observation of numerous treatments while simultaneously permitting new therapeutic agents to enter and leave the study without having to modify or halt the ongoing trial. Since the trial began, more than 14 new treatments have been completed for the management of breast cancer, with many more single agents or combinations of therapeutics being planned or tested [58,69]. The structure of the I-SPY2 trial has vastly driven the field of personalized treatment for breast cancer and has broken the status quo for clinical trial structure. When designing clinical trials in the future, clinicians and researchers must consider the I-SPY2 trial structure to evaluate if a platform trial would benefit patients and study outcomes.

### 4.5. Recently Completed Immunotherapy Clinical Trials for BC

While there are extensive ongoing immunotherapy clinical trials for BC treatment, there are also a handful of recently completed immunotherapy clinical trials. The bulk of these trials utilize anti-PD-1/PD-L1 immune checkpoint inhibitors in combination with commonly used chemotherapy drugs. Overall, the majority of these trials examined PFS, OS, and pCR rates after chemotherapy treatment with or without immunotherapy. Selected immunotherapy clinical trials for the treatment of BC can be found in Table 5.

Most notably, the results from the KEYNOTE-522 randomized controlled phase III trial (NCT03036488) received FDA approval in 2021 for the anti-PD-1 ICI, pembrolizumab, in combination with chemotherapy before and after surgery [58,70,71,72]. The study evaluated 1174 early TNBC patients for the efficacy and safety of pembrolizumab with a standard chemotherapy regimen consisting of nab-paclitaxel, paclitaxel, gemcitabine, or carboplatin when compared to the placebo with chemotherapy [58,70,71]. Of the 1174 patients who underwent randomization, 784 were assigned to the pembrolizumab–chemotherapy group and 390 to the placebo–chemotherapy group [71]. The results indicate that for patients with early triple-negative breast cancer, neoadjuvant pembrolizumab plus chemotherapy, followed by adjuvant pembrolizumab after surgery, resulted in significantly longer event-free survival (84.5% at 36 months) than neoadjuvant chemotherapy alone (76.8% at 36 months) [58,71]. It should also be noted that adverse events occurred predominantly during the neoadjuvant phase and were consistent with the established safety profiles of pembrolizumab and chemotherapy [71]. Although the majority of immunotherapy clinical trials are for the treatment of TNBC, the GIADA phase II trial (NCT04659551) evaluated neoadjuvant chemo-endocrine therapy and immunotherapy for pre-menopausal luminal B BC patients [58,73]. A total of 43 patients were enrolled in the trial and treated with a combination of epirubicin and cyclophosphamide [58,73]. This was followed by the combination of nivolumab, an anti-PD-1 inhibitor, and triptorelin started concurrently with chemotherapy, and exemestane started in parallel with nivolumab [58,73]. A pCR was achieved for 7/43 patients, and the pCR rate was significantly higher for patients with PAM50 basal breast cancer (50%) compared with other subtypes (luminal A, 9.1%; luminal B, 8.3%) [73]. Therefore, the data generated from this clinical trial indicates that luminal B-like breast cancers with a basal molecular subtype and/or a state of immune activation may respond to sequential anthracyclines and anti-PD-1 [73].

In the past two decades, the monoclonal antibody trastuzumab has been the most successful for BC treatment [70,74,75]. Trastuzumab first received FDA approval in 1998 for the treatment of invasive and metastatic HER2+ BC [70,74,75]. Prior to FDA approval, HER2+ was associated with poor outcomes and higher mortality rates than other breast cancer subtypes, resulting in similar mortality rates as TNBC [70,74,75,76]. In 2001, a pivotal Phase III trial of 469 women showed that adding trastuzumab to standard chemotherapy (paclitaxel or anthracycline/cyclophosphamide) resulted in improved response rates (50% versus 32%), extended time to progression (7.4 months versus 4.6 months), and improvement in median overall survival (25 versus 20 months). The relative risk of death was also reduced by 20% at a median follow-up of 30 months, further promoting the use of trastuzumab in BC treatment [70,74,75,76]. Since trastuzumab, an additional immunotherapeutic agent pertuzumab has been developed and FDA-approved to treat patients with HER2+ disease [70,74,75,76].

## 5. Limitations

While immunotherapy has shown to be a promising cancer treatment, there are also limitations that must be addressed. Immune checkpoint inhibitors are the most commonly used immunotherapy but have been reported to induce morbidities that can affect the cardiovascular, endocrine, rheumatological, pulmonary, neurological, and hepatic systems, along with immune-related adverse events and occasionally death in numerous cohorts of cancer patients [77,78,79].

Immune checkpoint inhibitors have been associated with the development of various cardiovascular toxicities, including myocarditis, cardiomyopathy, pericarditis, and arrhythmias, with myocarditis acting as the most commonly (45%) observed cardiovascular immune-related adverse event [80]. Myocarditis is a rare but serious heart condition that is caused by inflammation of the heart muscle [80]. Overall, melanoma and lung cancer patients have been shown to have higher rates of myocarditis following immune checkpoint inhibitor treatment when compared to other cancer types [81,82,83]. The risk of myocarditis may differ between various classes of immune checkpoint inhibitors with anti-CTLA-4 monotherapy (3.3%), anti-PD-L1 (2.4%), and anti-PD-1 agents (0.5%) [83]. Interestingly, a case series for cardiovascular toxicities resulting from immune checkpoint inhibitors showed that more than 60% of patients had a pre-existing cardiac pathology or peripheral vascular disease, and most of them experienced immune-related adverse events involving other organ systems [80,84].

Neurological and neuromuscular complications have been associated with immune checkpoint inhibitors, specifically anti-PD-1 inhibitors [85,86,87]. Although neurological adverse events occur less than five percent of the time, their potential severity and consequent interruptions to cancer treatment make them of particular importance [88]. While the majority of neurological complications cause peripheral neuropathies, immunotherapy has also been associated with an increased risk of encephalitis and paraneoplastic disorders affecting the central nervous system [87,88]. A systematic review recently published in the *Journal of the American Medical Association* evaluated the use of immune checkpoint inhibitors to treat various malignant neoplasms [89]. Interestingly, the risk of neurological adverse events following immune checkpoint inhibitors was lower when compared with chemotherapy but higher when compared with the placebo [89]. Overall, the published clinical studies suggest patients treated with checkpoint inhibitors are less likely to develop neurologic adverse events compared with other cancer medications, particularly cytotoxic chemotherapy [89].

It is important to note that up to 60% of melanoma patients treated with immune checkpoint inhibitors develop severe immune-related adverse events [90]. Peripheral blood samples were analyzed from patients with melanoma treated with anti-PD-1 monotherapy or anti-PD-1 and anti-CTLA-4 combination [90,91]. Prior to treatment with immune checkpoint inhibitors, diverse CD4+ effector memory T cells present that have been shown to be associated with severe immune-related adverse event development [90,91]. While this is a drawback, oncologists are able to identify severe immune-related adverse effects associated with immune checkpoint inhibitors [92]. Additionally, corticosteroid treatment has been shown to be effective with the continued administration of immune checkpoint inhibitors [92].

Additional toxicities affecting the endocrine, rheumatological, pulmonary, and hepatic systems are also reported [93,94,95,96]. In rare cases, immune checkpoint inhibitors can result in fatal toxic effects. A meta-analysis of 112 clinical trials involving 19,217 patients treated with immune checkpoint inhibitors showed toxicity-related fatality rates for anti–PD-1 (0.36%), anti-PD-L1 (0.38%), anti-CTLA-4 (1.08%), and PD-1/PD-L1 plus CTLA-4 (1.23%) [77,79]. Although there is a risk of death associated with complications of immune checkpoint inhibitor therapy, it is within or well below fatality rates for common oncologic interventions such as platinum-doublet chemotherapy (0.9%), allogeneic stem cell transplant (15%), targeted therapy with angiogenesis or tyrosine kinase inhibitors (0%-4%), and complex oncology surgeries (1–10%) [77].

Finally, differences in the efficacy and safety of varying immune checkpoint inhibitors must be addressed. A systematic review comprising 21,261 patients evaluated the efficacy and safety of PD-1 and PD-L1 inhibitors for patients with solid tumors. The results indicate that nivolumab, pembrolizumab, atezolizumab, and durvalumab yielded equivalent survival, while avelumab was associated with unfavorable survival [97]. Additionally, PD-1/PD-L1 inhibitors were comparable in the terms of treatment-related risk and safer than conventional therapies [97]. While all clinically available TNBC PD-1/PD-L1 inhibitors are administered intravenously, they yield varying efficacy in terms of OS, PFS, and pCR [98,99]. A systematic review of 5324 patients examined the comparative efficacy and safety of PD-1/PD-L1 inhibitors for the treatment of TNBC [99]. Pembrolizumab, a PD-1 inhibitor, was found to have a pooled OS of 0.82, PFS of 0.82, and a pCR of 2.79, whereas atezolizumab, a PD-L1 inhibitor, resulted in an OS of 0.92, PFS of 0.82, and a pCR of 1.94 in a neoadjuvant setting [99]. While the results from both inhibitors generate favorable outcomes, understanding the differences in efficacy is important to note.

## 6. Conclusions and Future Trends

The evolution of immunotherapy throughout the last century has propelled the field of targeted cancer treatment immensely. Within immunotherapeutic treatments, immune checkpoint inhibitors have proven to be safe, efficient, and effective, specifically for the treatment of breast cancer. Immunotherapy may be the answer to overcoming treatment challenges in TNBC patients who have previously been excluded from targeted therapies. Before exclusively relying on immunotherapy, we must improve therapeutic approaches, such as identifying biomarkers, overcoming adverse side effects, and developing a further understanding of the tumor microenvironment. When looking for future clinical practices, an in-depth evaluation of the I-SPY2 clinical trial will broaden our understanding of innovative therapies for breast cancer patients. Additionally, genetically engineered CAR-Ts have shown clinical promise, specifically for the treatment of HER2-enriched breast cancer. Understanding both benchtop and bedside immunotherapy research will allow for vast developments in breast cancer treatment.

## Figures and Tables

**Table 1:** Breast cancer subtype classification. Five major subtypes of breast cancer characterized by hormone status, HER2 status, Ki67 status, and overall outcome and prevalence.

Subtype	Hormone Status	HER2 Status	Ki67 Status	Outcome	Prevalence
Luminal A	[ER+] [PR+]	−	−	Good	40%
Normal-like	[ER+] [PR+]	−	−	Intermediate	5–8%
Luminal B	[ER+] [PR+]	+/−	+	Poor/ intermediate	20%
HER2-enriched	[ER−] [PR−]	+	+	Poor	10–15%
Triple negative	[ER−] [PR−]	−	+	Poor	15–20%

**Table 2:** Selected immunotherapy clinical trials in HR-positive breast cancer.

Identifier Number	Phase	Status and End Date	Participants	Treatment Type	Immunotherapeutic Agent	Joint Treatment
NCT04148937	I	Completed May 2021	150	Anti-PD-1/PD-L1	Pembrolizumab	LY3475070
NCT04360941	I	Ongoing August 2025	45	Anti-PD-1/PD-L1	Avelumab	Palbociclib
NCT05187338	I/II	Ongoing October 2024	100	Anti-CTLA-4	Ipilimumab	Pembrolizumab plus durvalumab
NCT05620134	I/II	Ongoing February 2026	149	Anti-CTLA-4	JK08 (CTLA-4 targeting IL-15 antibody fusion protein)	None
NCT05203445	II	Ongoing January 2026	23	Anti-PD-1/PD-L1	Pembrolizumab	Olaparib
NCT02971748	II	Ongoing December 2024	37	Anti-PD-1/PD-L1	Pembrolizumab	HR therapy, radiation
NCT02957968	II	Ongoing February 2025	47	Anti-PD-1/PD-L1	Pembrolizumab	Decitabine, followed by cyclophosphamide, paclitaxel, carboplatin
NCT04683679	II	Ongoing January 2025	56	Anti-PD-1/PD-L1	Pembrolizumab	Olaparib, radiation
NCT04443348	II	Ongoing December 2024	120	Anti-PD-1/PD-L1	Pembrolizumab	Radiation therapy boost, paclitaxel, carboplatin, cyclophosphamide, doxorubicin, capecitabine
NCT04243616	II	Ongoing January 2025	36	Anti-PD-1/PD-L1	Cemiplimab	Paclitaxel, carboplatin, doxorubicin, cyclophosphamide
NCT01042379	II	Ongoing December 2031	5000	Anti-PD-1/PD-L1	Cemiplimab	40 different treatments
NCT01042379	II	Ongoing December 2031	5000	Anti-PD-1/PD-L1	Durvalumab	Olaparib
NCT03650894	II	Ongoing April 2025	138	Anti-CTLA-4	Ipilimumab	Nivolumab, bicalutamide
NCT02491697	II	Ongoing August 2030	400	Dendritic cell therapy	DC-CIK immunotherapy	CIK

**Table 3:** Selected immunotherapy clinical trials in HER2-positive breast cancer.

Identifier Number	Phase	Status and End Date	Participants	Treatment Type	Immunotherapeutic Agent	Joint Treatment
NCT04360941	I	Ongoing December 2025	45	Anti-PD-1/PD-L1	Avelumab	Palbociclib
NCT04511871	I	Ongoing March 2025	15	CAR-T therapy	CCT303-406 CAR-modified autologous T cells (CCT303-406)	None
NCT03696030	I	Ongoing February 2025	39	CAR-T therapy	HER2-CAR T cells	None
NCT03740256	I	Ongoing December 2038	45	CAR-T therapy	HER2-CAR T cells	CAdVEC oncolytic virus
NCT02063724	I	Completed September 2022	15	Dendritic cell therapy	HER2 pulsed DC vaccine	None
NCT02061423	I	Completed December 2023	7	Dendritic cell therapy	HER2 pulsed DC vaccine	None
NCT03387553	I	Ongoing August 2026	31	Dendritic cell therapy	DC vaccine (DC1)	None
NCT04319757	I	Ongoing June 2024	36	Natural killer cell therapy	ACE1702	None
NCT04278144	I/II	Ongoing October 2026	390	Anti-PD-1/PD-L1	Nivolumab	BDC-1001
NCT01042379	II	Ongoing December 2031	5000	Anti-PD-1/PD-L1	Cemiplimab	40 different treatments
NCT01042379	II	Ongoing December 2031	5000	Anti-PD-1/PD-L1	Durvalumab	Olaparib
NCT04348747	II	Ongoing April 2025	23	Dendritic cell therapy	Anti-HER2/HER3 DC vaccine	Anti-PD1, IFNa2b

**Table 4:** Selected immunotherapy clinical trials for triple-negative breast cancer.

Identifier Number	Phase	Status and End Date	Participants	Treatment Type	Immunotherapeutic Agent	Joint Treatment
NCT05422794	I	Ongoing December 2025	57	Anti-PD-1/PD-L1	Pembrolizumab	ZEN003694, nab-paclitaxel
NCT03362060	I	Ongoing December 2025	20	Anti-PD-1/PD-L1	Pembrolizumab	PVX-410 vaccine
NCT04427293	I	Ongoing July 2026	12	Anti-PD-1/PD-L1	Pembrolizumab	Lenvatinib
NCT03720431	I	Completed October 2022	11	Anti-PD-1/PD-L1	Pembrolizumab	TTAC-0001
NCT02977468	I	Ongoing December 2024	15	Anti-PD-1/PD-L1	Pembrolizumab	Intraoperative radiation
NCT04265872	I	Ongoing December 2024	20	Anti-PD-1/PD-L1	Pembrolizumab	Bortezomib plus cisplatin injections; bortezomib followed by pembro/cis
NCT03310957	I/II	Ongoing December 2024	211	Anti-PD-1/PD-L1	Pembrolizumab	Ladiratuzumab vedotin
NCT02752685	II	Ongoing December 2024	70	Anti-PD-1/PD-L1	Pembrolizumab	Nab-paclitaxel
NCT05681728	II	Ongoing June 2024	26	Anti-PD-1/PD-L1	Pembrolizumab	Paclitaxel, cyclophosphamide, epirubicin
NCT04683679	II	Ongoing January 2025	56	Anti-PD-1/PD-L1	Pembrolizumab	Olaparib, radiation
NCT04427293	II	Ongoing July 2026	29	Anti-PD-1/PD-L1	Pembrolizumab	AE37 peptide vaccine
NCT04191135	II	Ongoing September 2024	460	Anti-PD-1/PD-L1	Pembrolizumab	Olaparib, carboplatin, gemcitabine
NCT02768701	II	Completed May 2023	40	Anti-PD-1/PD-L1	Pembrolizumab	Cyclophosphamide
NCT03121352	II	Completed May 2022	30	Anti-PD-1/PD-L1	Pembrolizumab	Nac-paclitaxel, carboplatin
NCT03567720	II	Ongoing September 2024	65	Anti-PD-1/PD-L1	Pembrolizumab	Tavokinogene telseplasmid, immunopulse, nab-paclitaxel
NCT04468061	II	Ongoing April 2027	110	Anti-PD-1/PD-L1	Pembrolizumab	Sacituzumab govitecan
NCT02755272	II	Ongoing April 2025	87	Anti-PD-1/PD-L1	Pembrolizumab	Carboplatin, gemcitabine
NCT04230109	II	Ongoing October 2026	51	Anti-PD-1/PD-L1	Pembrolizumab	Sacituzumab govitecan
NCT04443348	II	Ongoing December 2024	120	Anti-PD-1/PD-L1	Pembrolizumab	Radiation therapy boost, paclitaxel, carboplatin, cyclophosphamide, doxorubicin, capecitabine
NCT02819518	III	Completed October 2023	882	Anti-PD-1/PD-L1	Pembrolizumab	Nab-paclitaxel, paclitaxel, gemcitabine, carboplatin
NCT02393794	I/II	Ongoing July 2025	51	Anti-PD-1/PD-L1	Nivolumab	Romidepsin, cisplatin
NCT04331067	I/II	Ongoing May 2026	50	Anti-PD-1/PD-L1	Nivolumab	Paclitaxel, carboplatin, cabiralizumab
NCT03487666	II	Completed December 2022	45	Anti-PD-1/PD-L1	Nivolumab	Capecitabine
NCT04159818	II	Ongoing December 2026	52	Anti-PD-1/PD-L1	Nivolumab	Cisplatin, doxorubicin (low dose)
NCT02499367	II	Ongoing August 2025	84	Anti-PD-1/PD-L1	Nivolumab	Radiation, doxorubicin (low dose), cyclophosphamide, cisplatin
NCT03818685	II	Ongoing May 2024	114	Anti-PD-1/PD-L1	Nivolumab	Ipilimumab, capecitabine
NCT01042379	II	Ongoing December 2031	5000	Anti-PD-1/PD-L1	Cemiplimab	40 different treatments
NCT01042379	II	Ongoing December 2031	5000	Anti-PD-1/PD-L1	Durvalumab	Olaparib
NCT03498716	III	Completed August 2023	2300	Anti-PD-1/PD-L1	Atezolizumab	Paclitaxel, doxorubicin/epirubicin, cyclophosphamide
NCT04111510	II	Completed January 2023	6	Tumor-infiltrating lymphocyte (TIL) therapy	TIL LN-145	None
NCT04348747	II	Ongoing April 2025	23	Dendritic cell therapy	Anti-HER2/HER3 DC vaccine	Anti-PD1, IFNa2b

**Table 5:** Recently completed immunotherapy clinical trials for breast cancer.

Identifier Number	Phase	Status and End Date	Participants	Treatment Type	Drugs Used	Primary Results
NCT02425891	III	Completed August 2021	902	Anti-PD-1/PD-L1	Atezolizumab +/− nab-paclitaxel	ITT:PFS: 7.2 vs. 5.5 mo, HR = 0.80 ITT:OS: 21.0 vs. 18.7 mo, HR = 0.87 PD-L1+ PFS: 7.5 vs. 5.2 mo, HR = 0.63 PD-L1+ OS: 25.4 vs. 19.7 mo HR = 0.69
NCT03036488	III	Completed/ ongoing September 2025	1174	Anti-PD-1/PD-L1	Pembrolizumab +/− nab-paclitaxel, paclitaxel, gemcitabine, carboplatin	PFS: 7.5 vs. 5.6 mo HR = 0.82 CPS ≥ 10: PFS: 9.7 vs. 5.6 mo (hierarchical) HR = 0.66 OS: 23 vs. 16.1 mo HR = 0.73 CPS ≥ 1: PFS: 7.6 vs. 5.6 mo HR = 0.75, FDA-Approved
NCT02555657	III	Completed November 2020	1098	Anti-PD-1/PD-L1	Pembrolizumab +/− capecitabine, eribulin, gemcitabine, vinorelbine	ITT:PFS: 2.1 vs. 3.3 mo. HR = 1.60 ITT:OS:9.9 vs. 10.8 mo, HR = 0.97 PD-L1+:PFS:n2.1 vs. 4.3 mo, HR = 1.14 PD-L1+:OS: 12.7 vs. 11.6 mo, HR = 0.78
NCT02129556	I/II	Completed April 2017	58	Anti-PD-1/PD-L1	Pembrolizumab and trastuzumab	Pembrolizumab +trastuzumab was safe and showed activity and durable clinical benefit in patients with PD-L1-positive, trastuzumab-resistant, advanced, HER2-positive breast cancer (*Lancet*).
NCT02924883	II	Completed February 2020	202	Anti-PD-1/PD-L1	Atezolizumab, trastuzumab emtansine, placebo	PFS: 8.2 vs. 6.8 8 mo HR = 0.82 PD-L1 + PFS: 8.5 vs. 4.1 mo HR+: HR: 1.08 HR−: HR: 0.58
NCT04659551	II	Completed May 2020	43	Anti-PD-1/PD-L1	Epirubicin, cyclophosphamide, triptorelin, exemestane, nivolumab	pCR: 16.3% (7.4–34.9) Luminal B-like breast cancers with a basal molecular subtype and/or a state of immune activation may respond to sequential anthracyclines and anti-PD-1.
NCT03036488	III	Completed/ ongoing September 2025	1774	Anti-PD-1/PD-L1	Carboplatin, paclitaxel, 4xAC +/− pembrolizumab	pCR: 64.8 vs. 51.2% PD-L1+ pCR: 68.9 vs. 54.9%
NCT01042379	II	Completed/ ongoing August 2025	181	Anti-PD-1/PD-L1	Paclitaxel followed by anthracycline-cyclophosphamide +/− pembrolizumab	pCR rates: HER2−: 44 vs. 17% HR+ and HER2−: 30 vs. 13% TNBC: 60 vs. 22%

## Abbreviations

HR	Hormone receptor
ER	Estrogen receptor
PR	Progesterone receptor
HER2	Human epidermal growth factor 2
TNBC	Triple-negative breast cancer
CAR-T	Chimeric antigen T Cell
CTLA-4	Cytotoxic T-lymphocyte antigen
PD-1	Programmed cell death protein 1
PD-L1	Programmed death-ligand 1
OS	Overall survival
DFS	Disease-free survival
ORR	Overall response rate
DCR	Disease control rate
PFS	Progression-free survival
ISAC	Immune-stimulating antibody conjugate
MTD	Maximum tolerable dose
